# Psilocybin-induced changes in cerebral blood flow are associated with acute and baseline inter-individual differences

**DOI:** 10.1038/s41598-023-44153-z

**Published:** 2023-10-14

**Authors:** Nathalie M. Rieser, Ladina P. Gubser, Flora Moujaes, Patricia Duerler, Candace R. Lewis, Lars Michels, Franz X. Vollenweider, Katrin H. Preller

**Affiliations:** 1 Department of Psychiatry, Psychotherapy and Psychosomatics, Psychiatric University Hospital Zurich, Lenggstrasse 31, Zurich, Switzerland; 2 Department of Psychiatry, Yale University School of Medicine, New Haven, CT 06510 USA; 3 School of Life Sciences, Arizona State University, Tempe, AZ 85281 USA; 4 Department of Neuroradiology, University Hospital Zurich, Zurich, Switzerland; 5 Neuroscience Center Zurich, University of Zurich and Swiss Federal Institute of Technology Zurich, Zurich, Switzerland

**Keywords:** Neuroscience, Human behaviour, Pharmacology

## Abstract

Research into the use of psilocybin for the treatment of psychiatric disorders is a growing field. Nevertheless, robust brain–behavior relationships linking psilocybin-induced brain changes to subjective drug-induced effects have not been established. Furthermore, it is unclear if the acute neural effects are dependent on individual heterogeneity in baseline characteristics. To address this, we assessed the effects of three oral doses of psilocybin vs. placebo on cerebral blood flow (CBF) using arterial spin labeling in healthy participants (N = 70; n = 31, 0.16 mg/kg; n = 10, 0.2 mg/kg; n = 29, 0.215 mg/kg). First, we quantified psilocybin-induced changes in relative and absolute CBF. Second, in an exploratory analysis, we assessed whether individual baseline characteristics and subjective psychedelic experience are associated with changes in CBF. Psychological and neurobiological baseline characteristics correlated with the psilocybin-induced reduction in relative CBF and the psilocybin-induced subjective experience. Furthermore, the psilocybin-induced subjective experience was associated with acute changes in relative and absolute CBF. The results demonstrated that inter-individual heterogeneity in the neural response to psilocybin is associated with baseline characteristics and shed light on the mechanisms underlying the psychedelic-induced altered state. Overall, these findings help guide the search for biomarkers, paving the way for a personalized medicine approach within the framework of psychedelic-assisted therapy.

## Introduction

The classic psychedelic psilocybin (4-phosphoryloxy-*N*,*N*-dimethyltryptamine) is a preferential serotonin (5-HT) 2A/1A receptor agonist^1^ that induces an altered state of consciousness including visual alterations, audio-visual synesthesia, experience of unity, or insightfulness^2^. 5-HT receptors mediate a variety of psychological and physiological processes, such as mood, memory, sensory perception, appetite, and sleep^3, 4^. Accordingly, the effects of psilocybin on human perception and state of consciousness are manifold. Psychedelic substances, psilocybin in particular, are currently intensively investigated for their potential in the treatment of psychiatric disorders, such as depression (e.g.^5–8^), anxiety and depression in terminal illness (e.g.^9, 10^), and addiction (e.g.^11^) with promising preliminary results. However, the underlying psychological and neurobiological mechanisms of action are widely unknown.

An increasing number of studies are therefore exploring alterations in brain activity and connectivity induced by psychedelics, and aim to understand the neurobiological effects that may underlie the potential clinical efficacy and gain insight into the neural substrates that give rise to the subjectively experienced altered state of consciousness (e.g.^12–15^). Recent resting state functional magnetic resonance imaging (rsfMRI) studies point to altered information processing due to increased global brain functional connectivity in sensory areas and simultaneously decreased functional connectivity in associative regions^12, 16^.

Positron emission tomography (PET) has been used to map changes in glucose metabolism, revealing absolute increases in metabolism mainly in frontal regions, more specifically the frontomedial, frontolateral, anterior cingulate, and temporomedial cortex^17–19^. In line with this, an arterial spin labeling (ASL) study investigating the acute effects of psilocybin on relative cerebral blood flow (rCBF) showed increased rCBF in frontal and temporal regions and decreased rCBF in parietal regions and the insula^20^. rCBF is the CBF signal corrected for global brain perfusion, whereas absolute cerebral blood flow (aCBF) is not corrected for global perfusion. Without adjusting for global brain perfusion, widespread psilocybin-induced decreases in aCBF in healthy participants were observed under the acute influence of psilocybin in two studies^15, 20^. Lastly, LSD led to increased aCBF in the visual cortex in healthy participants, which was associated with complex visual imagery^21^.

Despite our emerging understanding of the effects of psilocybin on the human brain, it remains unknown whether these neurobiological alterations are dependent on individual baseline neurobiological and psychological characteristics and how they relate to the altered state of consciousness experienced by participants. Answering these questions is of particular importance to uncover the clinical mechanisms of action of psilocybin and inform the selection of patients who may benefit most from this therapeutic approach.

So far, low sample sizes have prevented the reliable detection of brain-behavior associations in neuroimaging studies investigating the acute effects of psychedelics. In this study, we therefore leverage the largest neuroimaging dataset to date collected under the acute influence of psilocybin, which includes 70 healthy participants assessed with ASL, to explore whether changes in CBF are associated with individual baseline characteristics, and the altered state of consciousness induced by psilocybin. ASL is a non-invasive MRI method, to quantify local tissue perfusion, which in turn allows for quantitative CBF evaluation^22, 23^. As CBF delivers both oxygen and glucose to neurons, CBF can partially be driven by neuronal activity^24^. Given the impact of global changes in pharmacological ASL studies, it became common practice to quantify CBF with and without a global covariate, i.e. rCBF and aCBF, respectively (e.g.^25, 26^). We hypothesize that (1) all three psilocybin doses will induce rCBF increases in frontal regions and decreases in parietal regions; (2) all three psilocybin doses will induce wide-spread reductions in aCBF; (3) individual baseline characteristics and self-reported altered state of consciousness are associated with acute changes in CBF after psilocybin administration; and (4) CBF following placebo is associated with subjective, psilocybin-induced altered state of consciousness.

## Materials and methods

### Study design

The data presented here were collected as part of three studies conducted at the Psychiatric University Hospital Zurich, Switzerland. Detailed study procedures are described in the corresponding publications^16, 27, 28^. A subsample of the current CBF data has been published previously^20^. In total N = 70 healthy, right-handed participants were included in the analysis (mean age = 25.33, SD = 3.91; female/male = 29/41; mean verbal IQ (assessed by MWT-B) = 112.04, SD = 12.19, see Table 1). Participants were healthy according to medical history, physical examination, blood analysis, electrocardiography, and urine test for drug use. Participants with present or a history of psychiatric disorders or first-degree relatives with major psychiatric disorders were excluded from the study. This was assessed using the MINI-International Neuropsychiatric Interview, a structured psychiatric interview^29^, and the Structured Clinical Interview for DSM-IV Axis II Personality Disorders^30^. Pregnancy was excluded by urine test. Participants with a lifetime psychedelic use of more than ten instances before study participation were excluded. In these randomized, double-blind, placebo-controlled, cross-over studies, participants received either placebo (n = 31, 100% lactose; n = 39, 179 mg mannitol and 1 mg aerosil) or oral psilocybin (n = 31, 0.16 mg/kg; n = 10, 0.2 mg/kg; n = 29, 0.215 mg/kg) in a counterbalanced order at least 10 days apart. All studies were approved by the Cantonal Ethics Committee of Zurich and conducted following the revised declaration of Helsinki (2000). All participants provided written informed consent statements prior to study participation.

**Table 1:** Demographics and psychological measurements.

	Total	0.16 mg/kg	0.2 mg/kg	0.215 mg/kg
Demographics				
N	70	31	10	29
Age (M, SD)	25.33 (3.91)	24.06 (3.22)	26.10 (2.33)	26.41 (4.66)
Sex (f/m)	29/41	11/20	6/4	12/17
Verbal IQ (M, SD)^a^	112.04 (12.19)	114.03 (11.43)	101.60 (11.31)	113.52 (11.81)
SCL-90-R				
Global severity score (M, SD)	0.22 (0.20)	0.25 (0.23)	0.18 (0.18)	0.20 (0.17)
Obsessiveness (M, SD)	0.39 (0.34)	0.42 (0.33)	0.39 (0.41)	0.36 (0.34)
Depressive symptoms (M, SD)	0.34 (0.34)	0.38 (0.37)	0.33 (0.48)	0.29 (0.25)
Paranoid thinking (M, SD)	0.24 (0.34)	0.20 (0.28)	0.17 (0.18)	0.30 (0.42)
5D-ASC				
∆ Global score (M, SD)	26.96 (16.72)	22.94 (14.77)	23.55 (17.58)	32.44 (17.39)
∆ Experience of unity (M, SD)	28.12 (27.75)	24.75 (27.20)	25.46 (32.48)	32.63 (27.02)
∆ Spiritual experience (M, SD)	12.81 (19.72)	11.06 (20.17)	8.57 (14.35)	16.14 (20.85)
∆ Blissful state (M, SD)	40.16 (30.66)	36.69 (30.41)	40.07 (35.37)	43.90 (29.93)
∆ Insightfulness (M, SD)	28.65 (29.20)	25.41 (28.10)	24.47 (31.04)	33.56 (30.05)
∆ Disembodiment (M, SD)	28.01 (29.15)	18.78 (22.34)	31.83 (31.50)	36.56 (32.62)
∆ Impaired control and cognition (M, SD)	17.94 (15.79)	16.90 (12.40)	12.80 (11.54)	20.83 (19.69)
∆ Anxiety (M, SD)	5.77 (12.14)	5.68 (13.69)	3.62 (4.28)	6.61 (12.41)
∆ Complex imagery (M, SD)	45.15 (33.17)	35.16 (32.72)	37.90 (29.98)	58.33 (31.10)
∆ Elementary imagery (M, SD)	47.06 (33.40)	37.49 (34.72)	38.30 (31.71)	60.31 (28.71)
∆ Audio-visual synesthesia (M, SD)	41.76 (38.50)	28.44 (31.85)	37.20 (40.49)	57.56 (39.69)
∆ Changed meaning of percepts (M, SD)	36.25 (29.65)	31.19 (26.94)	37.70 (37.98)	41.16 (29.52)

*N* sample size, *M* mean, *SD* standard deviation, *f* female, *m* male.

Results are depicted for all three groups in total and separately for each of the three different doses. Demographical information of included participants and verbal IQ measured with the German Mehrfachwahl-Wortschatz-Intelligenztest (MWT-B). For the assessment of the current psychopathological symptoms at baseline we used the Symptom Checklist 90-R (SCL-90-R). The scores for each scale, including the global score range from 0 to 4. Lastly, the Five Dimensional Altered States of Consciousness questionnaire (5D-ASC) was used to measure subjective experiences of an altered state of consciousness 360 min after substance administration. Scores for each subscale, including the global score range from 0 to 100.

^a^Assessed with the German verbal IQ questionnaire (MWT-B), ∆: ratings on Psilocybin session–Placebo session.

### Behavioral measures

#### Symptom checklist 90-R (SCL-90-R)

The SCL-90-R^31^ is a self-report measure, which assesses nine different symptom clusters of psychopathology; somatization, obsessive–compulsive, interpersonal sensitivity, depression, anxiety, hostility, phobic anxiety, paranoid ideation, and psychoticism. Participants rate each of the 90 items on a five-point scale (0 = not at all and 4 = extremely) according to symptom severity. The global score reflects the mean score of all reported symptoms. Participants completed the questionnaire at a screening visit, before the substance administration day. The cutoff global severity score characterizing a German healthy sample has been set to C = 0.57^32^. The mean global severity score in our sample was C = 0.22. As our sample consisted of healthy participants who reported low psychopathological scores, we here focus on the subscales that participants rated most highly: obsessiveness, depressive symptoms, and paranoid thinking.

#### Five dimension altered states of consciousness (5D-ASC) questionnaire

The 5D-ASC^33, 34^ consists of five dimensions (94 questions) and includes 11 subscales indexed from 43 individual visual analog items ranging from 0 to 100 (0 = not more than usual, 100 = much more than usual). The questionnaire retrospectively measures the subjective experience of an altered state of consciousness. It was completed 360 min post substance administration. In addition to the 11 subscales, we included the 5D-ASC global score, which reflects the mean score of all 94 items. The eleven subscales include: experience of unity, spiritual experience, blissful state, insightfulness, disembodiment, impaired control and cognition, anxiety, complex imagery, elementary imagery, audio-visual synesthesia, and changed meaning of percept.

### Neuroimaging data acquisition

Imaging was performed on a Philips Achieva 3.0 T whole-body scanner (Best, The Netherlands) and a 32-channel receive head coil. Anatomical images were acquired 60 min after drug administration followed by a resting-state BOLD scan (data reported:^16^) and the resting state pseudo-continuous ASL (pCASL) scan (80–100 min after drug administration).

High-resolution T1-weighted anatomical images were collected using a voxel size of 1 × 1 × 1 mm^3^ (n = 60) or 0.7 × 0.7 × 0.7 mm^3^ (n = 10). ASL data were acquired using a pCASL sequence with the following parameters: TR = 4400 ms; TE = 20 ms; FOV = 240 × 240 mm^2^; matrix size = 80, 23 slices with a voxel size = 3 × 3 ×  7 mm, and no gap; gradient echo single shot EPI; SENSE 2.5; post-labeling delay of 1525 ms; label duration: 1650 ms; number of dynamics: 60 (n = 39) or 50 (n = 31). One dynamic consisted of a control and a labeled image, resulting in a total scan time of 4 min 24 s (n = 39) or 3 min 40 s (n = 31).

### Image processing

Preprocessing and analysis were conducted using the ASLtoolbox^35^ and Statistical Parametric Mapping software (SPM8 and 12, Wellcome Trust Centre for Neuroimaging, UK implemented in Matlab, Math Works, Natick, MA). For each scan, the following preprocessing steps were conducted: realignment, smoothing (Gaussian at full-width-at-half-maximum (FWHM) of 6 × 6 × 14 mm^3^), perfusion-weighted image construction and calculation, and normalization to the Montreal neurological image (MNI) template space to allow group comparison. One participant was excluded as co-registration failed. In a next step, the six movement parameter (3 translation and 3 rotation) were used to calculate mean framewise displacement based on the Power’s method^36^. No participants were excluded from the subsequent analyses due to motion (framewise displacement > 0.32 mm/TR^37^). Equilibrium brain tissue magnetization (M0) images were recorded separately using the same parameters as described for the pCASL sequence, apart from the TR (10,000 ms).

### CBF quantification

In the next step, individual gray matter CBF values were calculated per scan and corrected for T2*decay^22^. Participants with T2*decay-corrected gray matter CBF values higher than 100 (ml/100 g/min) or lower than 40 (ml/100 g/min) were excluded (n = 3) from subsequent analyses^38^. This resulted in a final sample of N = 70. The gray matter CBF values were considered in each perfusion-weighted image calculation.

Two separate analyses were conducted to assess aCBF and rCBF. Perfusion-weighted difference images were calculated by simple subtraction of the label and control images (Mcontrol–Mlabel) and subsequently combined with the gray matter CBF values to generate aCBF (equivalent to global CBF in our previous publication^20^). To calculate the voxel wise rCBF, a brain mask was created to include all gray matter voxels using the Anatomical Automatic Labeling (AAL) atlas. Next, the mean gray matter CBF signal was calculated for each participant by averaging across voxels within the mask. Dividing the aCBF maps by this average signal resulted in the rCBF (e.g.^39, 40^).

### Statistical analysis

To assess whether dose impacts CBF outcome, we conducted three one-way ANOVAs to compare the effect of dose (0.6 mg/kg, 0.2 mg/kg, 0.215 mg/kg) on CBF (increased rCBF, decreased rCBF, decreased aCBF). In a first step, using the Levene’s test, we assessed the homogeneity of variances of the groups. The variances of the groups were equal (increased rCBF: F(1, 67) = 0.143, p = 0.867; decreased rCBF: F(1,67) = 0.671, p = 0.515; decreased aCBF: F(1,67) = 0.739, p = 0.481). The one-way ANOVAs revealed no statistically significant difference in rCBF and aCBF between different doses. More specifically; increased rCBF resulted in F(1,67) = 1.595; p = 0.211; decreased rCBF in F(1,67) = 2.387; p = 0.09, and decreased aCBF revealed F(1,67) = 0.236; p = 0.79. Therefore, dose was not taken into account in further analyses. The unthresholded difference maps between the participants receiving the low (0.16 mg/kg) and high dose (0.215 mg/kg) are presented in the Supplementary Fig. 1.

To assess whether body weight influences CBF, we ran a partial correlation between body weight and CBF, correcting for dose. In line with a previous publication^20^, which was based on a subsample of the current data, no relationship between body weight and rCBF, corrected for dose, was revealed (see Supplementary Fig. 2). However, we found a significant association between body weight and decreased aCBF, corrected for dose (r =  − 0.33, p = 0.005, see Supplementary Fig. 2). To further investigate this, we conducted a full brain multiple regression analysis on the effect of bodyweight on aCBF. The analysis showed no effect of bodyweight on aCBF (p_FWE_ < 0.05). Given these results together with a previous publication reporting no influence of body weight on pharmacokinetics^41^, body weight was not taken into account in the following analyses. Lastly, scatterplots reporting the association between dose, 5D-ASC global score, and CBF-changes are presented in Supplementary Fig. 3.

For the second level analysis, a paired sample t-test was applied to compare psilocybin vs. placebo conditions. We report significant changes with a threshold of p < 0.05 cluster-level FWE corrected with a primary threshold of p < 0.001, k > 250. To avoid the merging of clusters, a peak-level threshold of p < 0.05 (FWE-corrected) and k > 100 was used for aCBF. The eigenvalues from each significant cluster were exported (psilocybin-placebo) and Pearson-correlated with behavioral measures (SCL-90-R and 5D-ASC). Next, we investigated whether inter-individual variation in placebo CBF was related to the strength of psilocybin-induced changes in CBF. For placebo measures, the Oldham’s method (OH) was used to avoid the problem of mathematical coupling due to repeated measurement in the same participants^42^. To this end, we used the mean of the two measurements and their difference. Thus, we correlated the averaged psilocybin-induced increased rCBF, decreased rCBF, and decreased aCBF with the corresponding values following placebo ((placebo CBF + psilocybin CBF)/2), (OH). Lastly, we assessed the relationship between placebo CBF in clusters that were significantly altered by psilocybin and subjectively experienced altered state of consciousness following psilocybin using Pearson-correlations. R was used for statistical analyses of behavioral and neural correlations and the package corrplot (version 0.92) for the correlation matrices^43^. Correlations were considered significant at p < 0.05 (two-tailed). Given the exploratory nature of this correlation analysis, no correction for multiple comparisons was applied.

## Results

We first quantified psilocybin-induced changes in rCBF and aCBF. Next, we explored the association between altered CBF following psilocybin and (i) neurobiological (placebo CBF) and (ii) behavioral characteristics (psychological characteristics and acutely experienced altered state of consciousness). Lastly, we investigated the relationship between placebo CBF and subjectively experienced altered state of consciousness following psilocybin.

### Relative CBF

**Figure 1:**
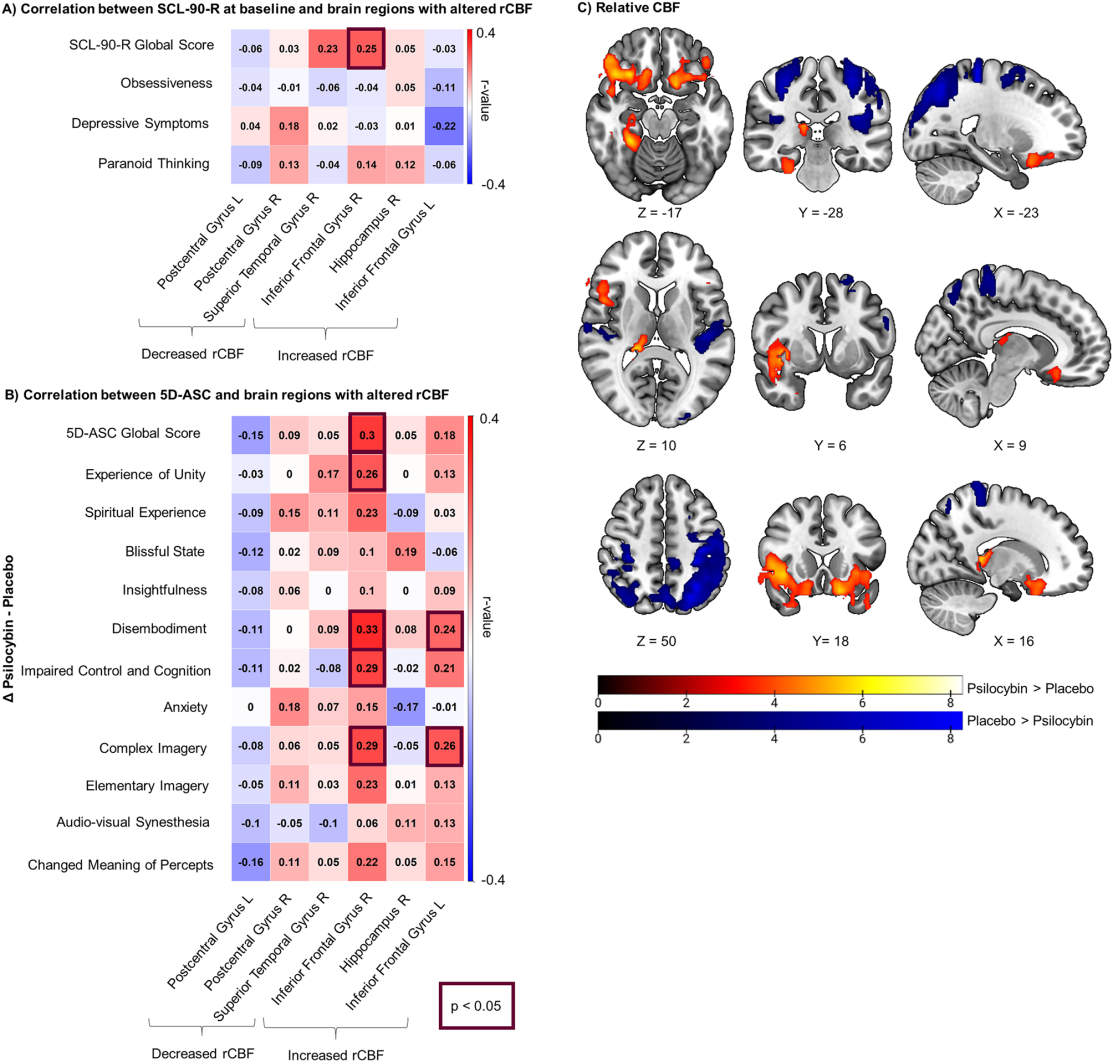
Associations between changes in relative cerebral blood flow (rCBF) (psilocybin-placebo) and behavior. (**A**) Correlation matrix demonstrating baseline SCL-90-R subscales and altered rCBF (psilocybin-placebo). (**B**) Correlation between the 11 subscales of the 5D-ASC difference score (psilocybin-placebo) and altered rCBF. (**C**) Brain maps illustrating rCBF in regions displaying significant increases (psilocybin > placebo) in red-yellow and rCBF in regions displaying significant decreases (placebo > psilocybin) in blue. Color scale shows t-scores. Pearson correlation. N = 70. SCL-90-R: Symptom checklist 90-R. 5D-ASC: Five dimension altered states of consciousness.

Psilocybin induced increases and decreases in rCBF (Fig. 1C). Increased rCBF following psilocybin (psilocybin > placebo) was observed in frontal (inferior frontal gyrus) and limbic regions (hippocampus, *p*_FWE-corrected_ < 0.05, detailed information about specific brain regions in Suppl. Table 1). Regions with decreased rCBF following psilocybin (placebo > psilocybin) were parietal (postcentral gyrus) and temporal (superior temporal gyrus, for detailed information see Suppl. Table 2). In the temporal cortex, both increased and decreased rCBF were observed. Specifically, rCBF was increased in the fusiform gyrus, while the superior temporal gyrus showed decreased rCBF.

### Absolute CBF

**Figure 2:**
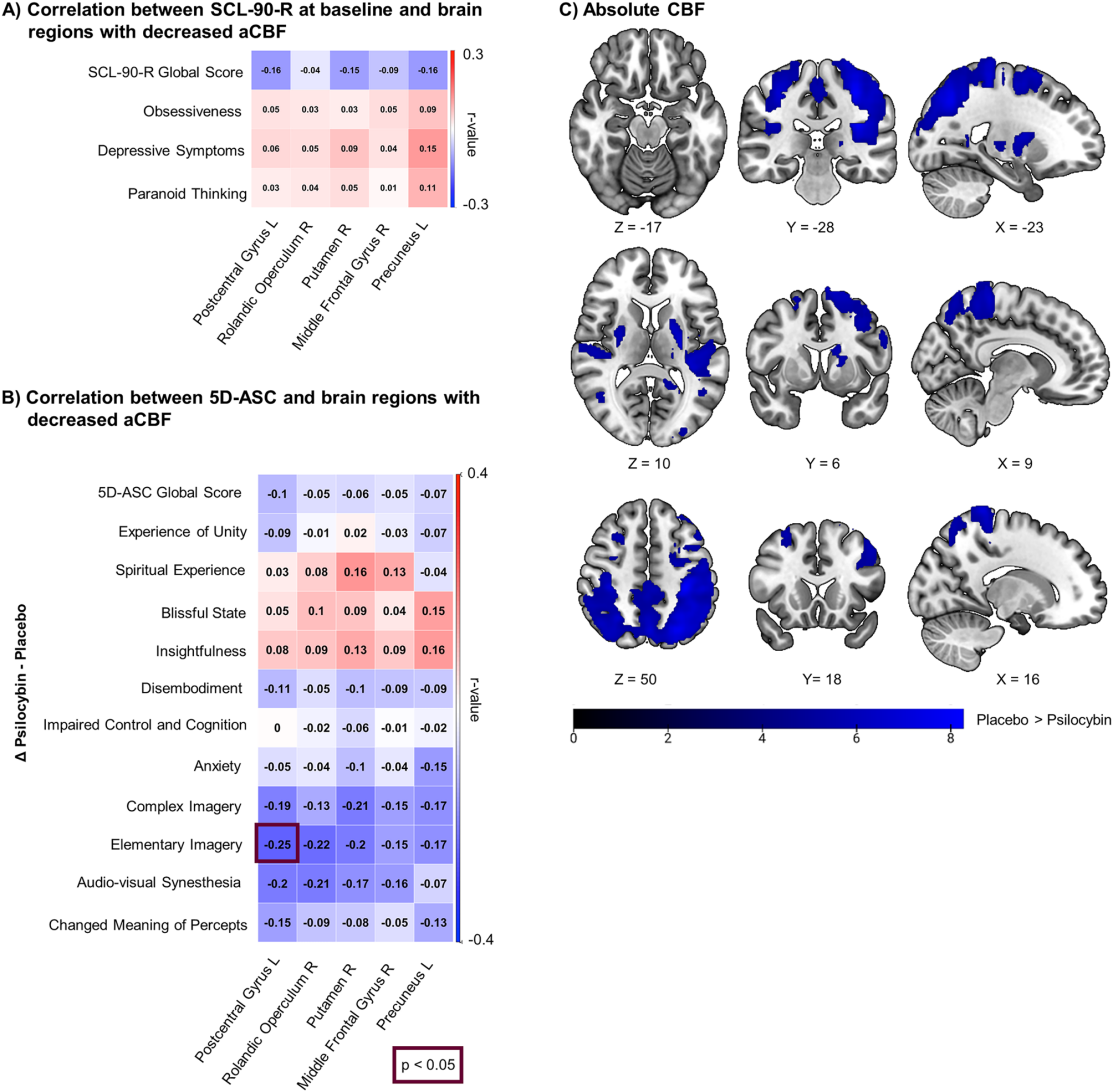
Associations between decreased absolute cerebral blood flow (aCBF) and behavior. (**A**) Correlation matrix showing association between baseline SCL-90-R subscales and aCBF in regions displaying significant decreases (psilocybin-placebo). (**B**) Correlation between the 11 subscales of the 5D-ASC difference score (psilocybin-placebo) and altered aCBF (psilocybin-placebo). (**C**) Brain maps presenting aCBF in regions displaying significant decreases (placebo > psilocybin). Color scale shows t-scores. Pearson correlation. N = 70. SCL-90-R: Symptom checklist 90-R. 5D-ASC: Five dimension altered states of consciousness.

The pCASL map, without adjusting for global brain perfusion, revealed significantly decreased aCBF in parietal (postcentral gyrus, rolandic operculum), limbic (putamen), and frontal regions (middle frontal gyrus (*p*_FWE-corrected_ < 0.05, for further information see Fig. 2C, Suppl. Table 3). We observed no psilocybin-induced increases in aCBF.

### Relationship between changes in CBF and baseline and acute behavioral measures

First, we explored the relationship between psilocybin-induced changes in rCBF and baseline psychological characteristics derived from the SCL-90-R, including the global score and the subscales obsessiveness, depressive symptoms, and paranoid thinking (Fig. 1A). While no subscale was linked to psilocybin-induced changes in CBF, we found a positive association between self-rated symptom global score and psilocybin-induced increased rCBF in the right inferior frontal gyrus (*r* = 0.25, *p* = 0.034). Specifically, participants with high global symptom levels showed strong increases in rCBF.

Second, we correlated the global score and the 11 subscales of the 5D-ASC with changes in rCBF. We found significant associations between the 5D-ASC global score, experience of unity, disembodiment, impaired control and cognition, and complex imagery and increased psilocybin-induced rCBF in the inferior frontal gyrus (see Fig. 1B). More specifically, the 5D-ASC global score was positively related to increased rCBF in the right inferior frontal gyrus (*r* = 0.3, *p* = 0.001). Participants who experienced stronger changes in the state of consciousness showed higher increases in rCBF. The subscale experience of unity revealed a similar pattern; i.e. participants who reported increased experience of unity showed increased rCBF in the right inferior frontal gyrus (*r* = 0.26, *p* = 0.029). One participant experienced strong impaired control and cognition. This was driving the significant positive correlation between impaired control and cognition and rCBF in the right inferior frontal gyrus (*r* = 0.29, *p* = 0.014). When excluding this participant, this correlation did not reach significance (*r* = 0.21,* p* = 0.09). Disembodiment and complex imagery revealed a similar pattern: disembodiment was associated with increased rCBF in the right (*r* = 0.33, *p* = 0.005) and left inferior frontal gyrus (*r* = 0.24, *p* = 0.048) with participants feeling stronger disembodiment showing stronger increases of rCBF in the inferior frontal gyrus. Complex imagery, too, was positively related to the right (*r* = 0.29, *p* = 0.016) and left inferior frontal gyrus (*r* = 0.26, *p* = 0.031).

For aCBF, we did not find any significant correlations with psychological baseline states (SCL-90-R) (see Fig. 2A). Acutely experienced elementary imagery was negatively correlated with reduced aCBF in the left postcentral gyrus (*r* =  − 0.25, *p* = 0.04, see Fig. 2B). Participants with higher self-reported elementary imagery showed a stronger decrease in aCBF in the postcentral gyrus. None of the other subscale-aCBF correlations reached significance.

### Relationship between placebo CBF and psilocybin-induced changes in CBF

**Figure 3:**
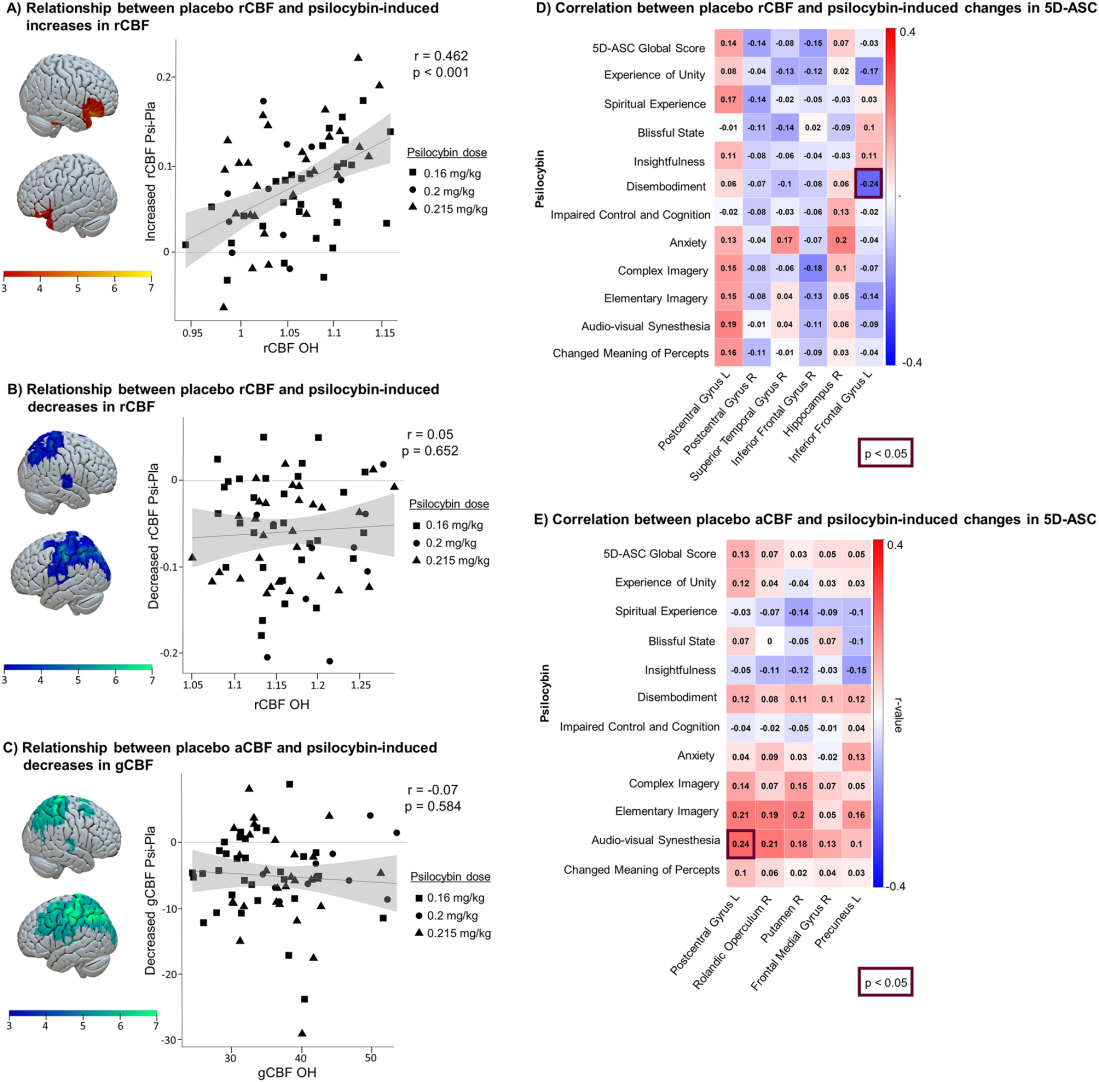
Relationship between baseline cerebral blood flow (CBF) and psilocybin-induced neural and subjective changes. (**A**) Psilocybin-induced rCBF in regions displaying significant increases (psilocybin-placebo) was significantly correlated with Oldham’s score [OH] ((placebo rCBF + psilocybin rCBF)/2) across participants. (**B**) Psilocybin-induced rCBF in regions displaying significant decreases (psilocybin-placebo) was not significantly correlated with Oldham’s score [OH] ((placebo rCBF + psilocybin rCBF)/2) across participants. (**C**) Psilocybin-induced aCBF in regions displaying significant decreases (psilocybin-placebo) was not significantly correlated with Oldham’s score [OH] ((placebo rCBF + psilocybin rCBF)/2) across participants. Scatterplots show the correlation between baseline CBF values (OH) and psilocybin-induced changes in CBF (psilocybin-placebo). Data points are symbol-coded to represent each subject at the psilocybin dose of 0.16 mg/kg, 0.2 mg/kg, 0.215 mg/kg). The gray background indicates the 95% confidence interval. D) Correlation between the 11 subscales of the 5D-ASC under the acute influence of psilocybin and placebo rCBF. E) Associations between the 11 subscales of the 5D-ASC under the acute influence of psilocybin and placebo aCBF, Pearson correlated. N = 70. 5D-ASC: Five dimension altered states of consciousness.

To explore whether neurobiological characteristics without pharmacological challenge are linked to psilocybin-induced changes in CBF, we quantified the association between placebo CBF and psilocybin-induced changes in CBF. Placebo rCBF values were positively associated with increased psilocybin-induced rCBF, namely, participants with higher rCBF in the inferior frontal gyrus and hippocampus in their regular waking state showed a stronger psilocybin-induced increase in rCBF (*r* = 0.46, *p* < 0.001) (Fig. 3A). Neither decreased rCBF (*r* = 0.05, *p* = 0.652) nor changes in aCBF (*r* = 0.07, *p* = 0.584) were associated with its placebo values (Fig. 3B,C).

### Relationship between placebo CBF and subjectively experienced altered state of consciousness

Lastly, to examine the association between placebo CBF and subjective experience following psilocybin administration, we correlated placebo CBF and the 11 subscales of the 5D-ASC (see Fig. 3D,E). Experienced disembodiment might be negatively associated with rCBF the left inferior frontal gyrus (*r* =  − 0.24,* p* = 0.049). Furthermore, audio-visual synesthesia was positively associated with placebo aCBF in the left postcentral gyrus (*r* = 0.24, *p* = 0.046), i.e. participants with higher placebo aCBF in this region reported higher psilocybin-induced audio-visual synesthesia.

## Discussion

Psilocybin is increasingly investigated in the treatment of psychiatric disorders (e.g.^6, 8, 9, 11, 44, 45^). To understand its mechanisms of action, various brain imaging studies have started to shed light on acute neurobiological changes induced by psilocybin (e.g.^12, 14–16, 46^). However, we still lack an understanding of how these acute changes relate to subjective drug effects. Furthermore, it is unclear whether individual baseline characteristics are related to the neurobiological effects induced by psilocybin. To help narrow these knowledge gaps, we leveraged the largest neuroimaging dataset to date collected under the acute influence of three different doses of psilocybin, including 70 healthy participants assessed with ASL. We investigated whether altered brain CBF is related to individual baseline characteristics and the psilocybin-induced altered state of consciousness. We show that: (1) psychological and neurobiological baseline characteristics are associated with psilocybin-induced increases in rCBF, (2) acute subjective effects correlate with changes in rCBF and aCBF, and (3) acute subjective experiences are linked to baseline CBF.

In line with a previous publication^20^, which was based on a subsample of the current data, we show that psilocybin acutely increases rCBF in frontal and limbic brain regions, and decreases rCBF mainly in parietal regions. In temporal regions, both increased and decreased rCBF was observed. Specifically, increased rCBF in temporal regions included the fusiform gyrus and decreased rCBF involved the superior temporal gyrus. Interestingly, psilocybin-induced changes in BOLD rsfMRI connectivity were reported in overlapping brain areas, namely hyperconnectivity in the left postcentral gyrus and right superior temporal gyrus^16^. While Preller et al.^16^ reported hyperconnectivity in these regions, the present study shows that psilocybin decreases rCBF in the same brain areas. Therefore, future studies should investigate whether alterations in rCBF are coupled with psilocybin-induced changes in the synchronization of brain regions.

Without adjusting for global brain perfusion, we found wide-spread decreases in aCBF in parietal, limbic, and frontal regions, which is in line with previous studies that administered psilocybin orally^20^ and intravenous^15^. Regressing out global signal perfusion as a confound or nuisance variable in fMRI analyses addresses artefacts such as head motion, respiration, cardiac rhythms, and scanner-related artifacts^47–49^. Global signal removal remains an ongoing debate, especially regarding resting-state BOLD data^50^. For pharmacological ASL it has become common practice to control for global signal and report analyses with and without a global signal covariate (e.g.^25, 26, 51^). Controlling for the global signal in ASL studies has been shown to improve reproducibility and signal-to-noise ratio in particular when measuring drug effects^40, 49, 52, 53^. This is in line with our current results, showing that rCBF is more sensitive to brain-behavior associations (see below).

To investigate whether neurobiological characteristics are associated with psilocybin-induced changes in CBF, we quantified the correlation between placebo CBF and changes in CBF induced by psilocybin. While we did not find any associations between placebo aCBF and psilocybin-induced changes in aCBF, placebo rCBF was positively correlated with increased psilocybin-induced rCBF. Specifically, participants with high rCBF in the frontal brain cortices and hippocampus in their regular waking state showed the strongest increase in rCBF under psilocybin. Therefore, baseline rCBF after placebo administration could be a predictive marker of the intensity of psilocybin-induced neuronal effects. This is in line with a recent study showing that placebo BOLD rsfMRI connectivity is associated with psilocybin-induced changes in connectivity^16^. Together, these results suggest that the effects of psilocybin may depend on measurable blood flow characteristics of the organization of each individual’s brain. While it is still unclear how acute psilocybin-induced effects map onto symptom improvements in clinical populations, in line with a personalized medicine approach, baseline rCBF should be explored as a predictive biomarker in clinical studies to identify patients most likely to benefit from psychedelic-assisted therapy.

In a next step, we explored the correlation between behavioral and neural effects. We found that the inferior frontal gyrus plays a key role in psilocybin-induced behavioral effects. We analyzed the association between baseline psychological state markers and acute changes in CBF. Self-rated psychological symptoms at baseline were correlated with psilocybin-induced increased rCBF in the right inferior frontal gyrus. Specifically, participants with higher levels of symptoms showed greater increases in rCBF. Although we investigated healthy participants who did not score high on these scales, it is possible that baseline psychological states may influence the acute reaction to psychedelics in patient populations.

Given that acute subjective psilocybin-induced effects have not consistently been linked to neural effects, next, we explored the correlation between changes in CBF and subjective alterations of consciousness. Increased rCBF in the inferior frontal gyrus was related to experience of unity, disembodiment, impaired control and cognition, and complex imagery. Several studies showed the involvement of the inferior frontal gyrus in language processing, motor control, and more recently also in the processing of empathy^54^. More specifically, the inferior frontal gyrus has been associated with emotion recognition, their evaluation, and emotional empathy^55^. Given that psychedelics lead to increased emotional empathy (e.g.^56–58^) and the consistent connection between inferior frontal gyrus and behavioral effects shown in this paper, the inferior frontal gyrus might be an underlying driving force for increasing emotional empathy by triggering disembodiment and visual imagery. However, the association between empathy, behavioral, and neural effects needs to be tested in future studies to gain understanding of the underlying processes.

Participants with increased self-reported experience of unity showed increased rCBF in the right inferior frontal gyrus. Experience of unity or feelings of unity is considered a subdimension of mystical-type experiences^59^. This type of experience has been reported by some participants after the administration of psychedelics. While experience of unity itself has not been shown to be related to therapeutic effects, the mystical-type experience has. Experiencing a strong psychological effect including insight, blissfulness, and feelings of unity has been associated with symptom decrease in depression, substance use disorder, and anxiety (i.e.^9, 60–63^). It is therefore possible that changes in activity of the inferior frontal gyrus contribute to clinical efficacy of psychedelics.

In addition, participants experiencing high visual imagery showed high psilocybin-induced rCBF in the right and left inferior frontal gyrus. Historically, visual alterations induced by psychedelic substances have mainly been associated with changes in visual networks^64^. More specifically, the intensity of visual alterations was linked to LSD-induced resting-state functional connectivity (i) within the primary visual cortex^21, 65^ and (ii) between the thalamus and the fusiform gyrus^46^. Increased aCBF in the visual cortex was associated with experiencing complex imagery after LSD administration^21^. Our results demonstrate that increased rCBF in frontal brain regions also contributes to visual alterations. The involvement of frontal regions in visual imagery has also been reported in previous studies. In particular, increased elementary imagery was associated with reduced BOLD activation in the left superior frontal gyrus and right precentral gyrus in response to a Go/No-Go task^66^. Furthermore, elementary imagery was negatively associated with aCBF in the left postcentral gyrus. Participants reporting stronger visual changes showed a stronger decrease of aCBF in this region. Alterations in activity and connectivity of the postcentral gyrus have repeatedly been reported after the administration of psychedelic substances (e.g.^16, 66, 67^), however, they have not been linked to psychedelic-induced visual alterations, yet.

From a psychiatric perspective, patients with addiction, major depression, or anxiety often exhibit an imbalance of visual imagery, such as diminished positive imagery and reduced vividness^68–70^. The subsequent elicited negative emotions associated with imagery strengthen maladaptive behavior patterns such as rumination, avoidance, and suppression^70^.

The beneficial effects of therapeutic approaches that target visual imagery in clinical settings underpin the importance of imagery in psychiatric disorders, i.e. therapeutic approaches that include imagery rescripting or promoting positive imagery^68, 70–72^. As psychedelics consistently induce visual alterations, strengthening visual imagery during and after the acute experience may increase its therapeutic potential.

In addition, participants reporting higher ratings of disembodiment showed increased rCBF in the left and right inferior frontal gyrus. The inferior frontal gyrus has been discussed in heautoscopy, which is the reduplication of one’s body, meaning to see one’s body at a distance^73^. Furthermore, lesion studies revealed its relation to somatoparaphrenia, where a patient beliefs that a limb does not belong to their body^74, 75^. Therefore, our findings are coherent with the notion of the inferior frontal gyrus being involved in bodily awareness. Lastly, we investigated whether subjective psilocybin-induced effects are related to inter-individual differences in CBF without pharmacological challenge. Placebo CBF was associated with acutely experienced, psilocybin-induced disembodiment and audio-visual synesthesia. In combination with the finding above, both baseline and acute rCBF in the inferior frontal gyrus were associated with psilocybin-induced disembodiment. Therefore, experiencing disembodiment might be partially driven by baseline neural characteristics.

Audio-visual synesthetic experiences can occur without any intervention in some people or can be induced experimentally^76^. Psychedelics often induce audio-visual synesthesia even in participants who are not experiencing any synesthesia during their regular conscious state (e.g.^77^). The underlying mechanisms, however, remain largely unknown. One explanation suggests that drug-induced synesthesia is based on the destabilized thalamic projections of sensory inputs leading to an inaccurate integration of multisensory stimuli^78^. The second explanation is based on the assumption that non-synaesthetes suppress other sensory senses that are associated with the stimuli. Following psychedelics, the inhibition of other senses may be reversed by a decreased attentional discrimination towards incoming stimuli and induce the experienced synesthesia^79, 80^. Our results suggest that the strength of experiencing audio-visual synesthesia following psilocybin administration may be explained by inter-individual differences in the baseline organization of the individual’s brain. Given that parietal structures including the postcentral gyrus—which was also related to synesthesia under psilocybin in this study—have been identified to differ functionally between audio-visual synaesthetes and matched controls^81^, it is possible that individuals who are more prone to experiencing synesthesia also have stronger psilocybin-induced synesthetic effects.

The following limitations need to be acknowledged: Given the exploratory nature of the study, we did not correct our analyses for multiple comparisons. Furthermore, the correlational analysis does not allow for directional interpretation of the effects. Therefore, this work serves as a basis for future targeted, hypothesis-driven investigations. Lastly, we are evaluating single measurements of baseline and psilocybin conditions. Intra-individual variations may affect results and repeated measurement of both placebo and psilocybin conditions may increase reliability and reproducibility.

The present study replicated previous findings of psilocybin-induced changes in CBF^15, 20^ and demonstrated that baseline psychological symptoms and rCBF are associated with a psilocybin-induced increases in rCBF. Furthermore, acutely experienced feelings of unity, disembodiment, and visual imagery were correlated with altered rCBF and aCBF following psilocybin. In addition, variation in placebo CBF was associated with the strength of psilocybin-induced disembodiment and visual-auditory synesthesia. These results help to narrow down key knowledge gaps in our understanding of the relationship between behavioral measures and psilocybin-induced alterations in brain CBF, and thus point to potential mechanisms of action of psychedelics and baseline CBF as a potential biomarker for clinical efficacy. Understanding the mechanism of action is particularly important for clinical development as this knowledge can guide the therapeutic approach and pave the way for personalized medicine within the framework of psychedelic-assisted therapy.

### Supplementary Information

Supplementary Information.

## Data Availability

The datasets analysed in the current study are available from the corresponding author upon reasonable request.
