# Supplementary material for: Psilocybin-induced changes in cerebral blood flow are associated with acute and baseline inter-individual differences
Source: Sci Rep. 2023 Oct 14;13:17475. doi: 10.1038/s41598-023-44153-z (PMC10576760; doi:10.1038/s41598-023-44153-z)
Supplement: Supplementary file 1 — Supplementary Information. [file 41598_2023_44153_MOESM1_ESM.pdf]

Psilocybin-induced changes in cerebral blood flow are associated with acute and baseline inter-individual differences

Nathalie M. Rieser[1,*], Ladina P. Gubser[1], Flora Moujaes[1,2], Patricia Duerler[1], Candace R. Lewis[3], Lars Michels[4], Franz X. Vollenweider[1,+], and Katrin H. Preller[1,+]

[1]Department of Psychiatry, Psychotherapy and Psychosomatics, University Hospital of Psychiatry Zurich, Lenggstrasse 31, Zurich, Switzerland
[2]Department of Psychiatry, Yale University School of Medicine, New Haven, CT 06510, United States
[3]School of Life Sciences, Arizona State University, Tempe, AZ 85281, USA
[4]Department of Neuroradiology, University Hospital Zurich, Zurich, Switzerland, Neuroscience Center Zurich, University of Zurich and Swiss Federal Institute of Technology Zurich, Zurich, Switzerland

*Corresponding author: nathalie.rieser@bli.uzh.ch
+ equal contribution

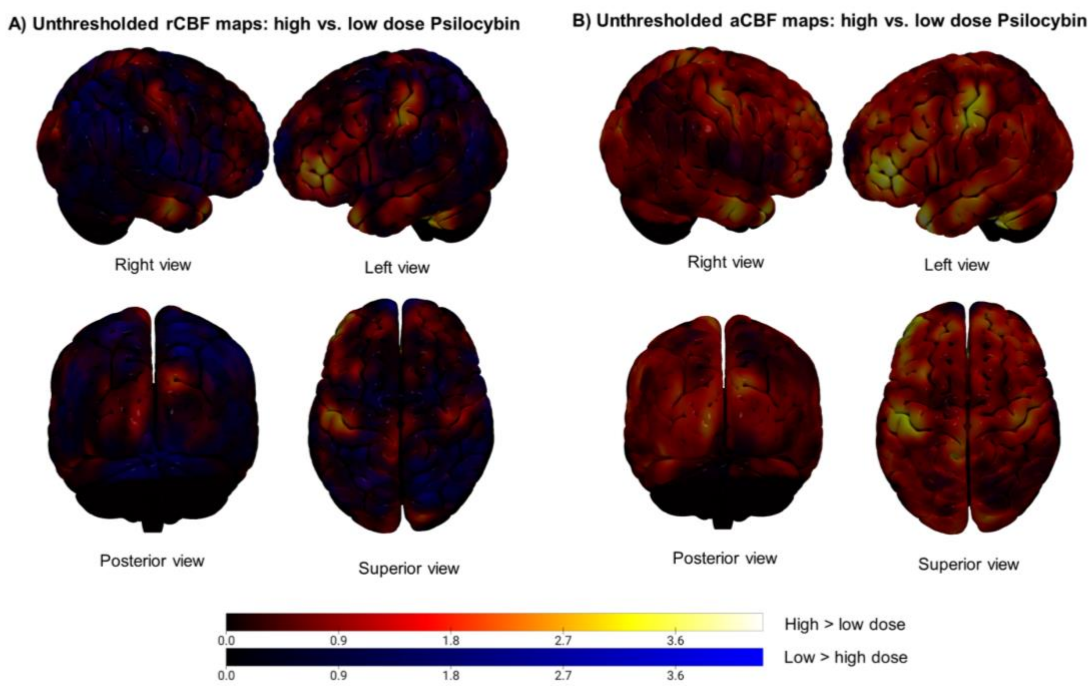

Supplementary Figure 1: Comparison between high and low dose psilocybin effects on CBF. A) Unthresholded t-score map showing the high (n=31, 0.215 mg/kg) vs. low dose n (n=29, 0.16 mg/kg) comparison (t-test) for relative CBF (rCBF). B) Unthresholded t-score map displaying the comparison between high (0.215 mg/kg) vs. low dose (0.16 mg/kg) dose of psilocybin (t-test). CBF: Cerebral Blood Flow.

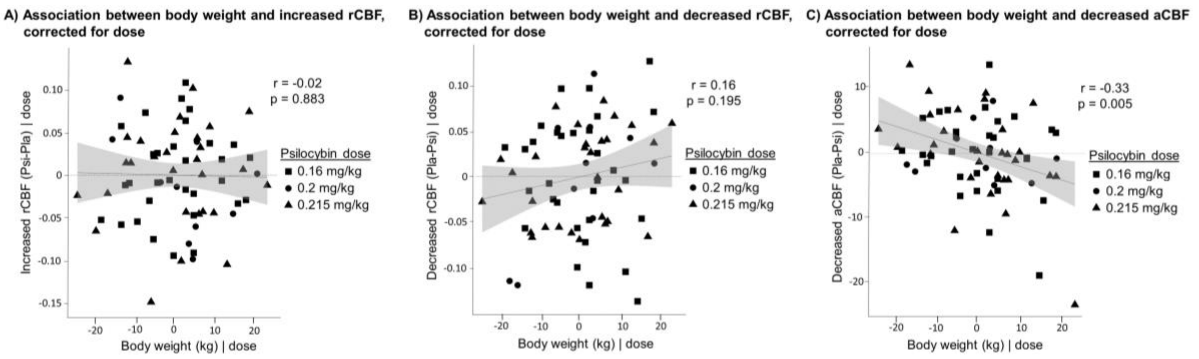

Supplementary Figure 2: Partial correlation between body weight and changes in CBF controlling for dose. A) Scatterplot displaying the partial correlation between body weight (in kg) and relative cerebral blood flow (rCBF) (psilocybin – placebo) in regions displaying significant increases, corrected for psilocybin dose (0.16 / 0.2 / 0.215 mg/kg); r=-0.01, p=0.883. B) Scatterplot showing the partial correlation between body weight and decreased rCBF (placebo - psilocybin) in regions displaying significant decreases, corrected for dose; r=0.16, p=0.195. C) Scatterplot showing the association between body weight and absolute cerebral blood flow (aCBF) (placebo - psilocybin) in regions displaying significant decreases, corrected for dose. The partial correlation coefficient is r=-0.33, p=0.005. Pearson correlation. CBF: Cerebral Blood Flow.

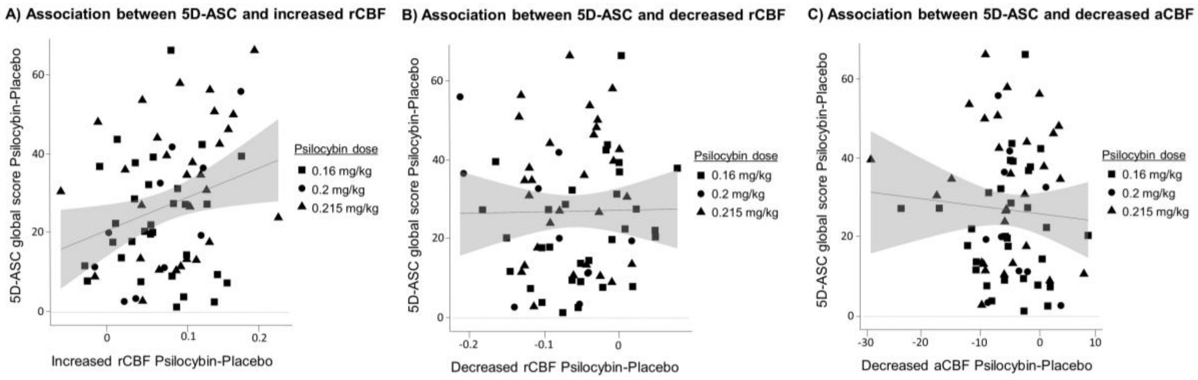

Supplementary Figure 3: Association between subjective effects and changes in CBF. A) Scatterplot representing the association between relative cerebral blood flow (rCBF) in regions displaying significant increases (psilocybin – placebo) and 5D-ASC global score color-coded for dose (0.16 mg/kg, 0.2mg/kg, 0.215mg/kg). B) Scatterplot displaying the relationship between rCBF (placebo - psilocybin) in regions displaying significant decreases and 5D-ASC global score symbol-coded for dose (0.16 mg/kg, 0.2mg/kg, 0.215mg/kg). C) The scatterplot displays the association between absolute cerebral blood flow (aCBF) in regions displaying significant decreases (placebo - psilocybin) and the 5D-ASC global score symbol-coded for dose (0.16 mg/kg, 0.2mg/kg, 0.215mg/kg). N=70. CBF: Cerebral Blood Flow; 5D-ASC: Five dimension altered state of consciousness questionnaire global score.

Table 1: Brain regions with increased relative cerebral blood flow

Cluster #	Brain Region (MNI)	Coordinates {mm}			cluster-level		peak-level		
		x	y	z	**k cluster size**	**p-value** (FWE-corr.)	***t***	***Z***	**p-value** (FWE-corr.)
**rCBF Psilocybin > Placebo**									
1	**Inferior Frontal Gyrus R**	48	32	-6	3852	< 0.000	6.72	5.87	< 0.000
	Medial Temporal Pole R	48	26	-36			5.97	5.34	0.003
	Inferior Frontal Gyrus R	30	24	-16			5.9	5.29	0.004
2	**Hippocampus R**	24	-38	-2	1589	< 0.000	6.66	5.83	< 0.000
	Fusiform Gyrus R	28	-40	-12			6.3	5.58	0.001
	Thalamus R	14	-28	14			5.22	4.78	0.042
3	**Inferior Frontal Gyrus L**	-34	40	-2	1819	< 0.000	5.86	5.26	0.005
	Inferior Frontal Gyrus L	-16	18	-22			5.7	5.14	0.008
	Inferior Frontal Gyrus L	-46	30	-6			5.31	4.85	0.031

Note: rCBF: Relative Cerebral Blood Flow; L: Left, R: Right

Significance threshold set at p<0.05 cluster-level family-wise-error (FWE) for multiple corrections at an initial voxel-level threshold of p (uncorrected)<0.001, and an extent threshold of k=250 voxels.

Table 2. Brain regions with decreased relative cerebral blood flow

Cluster #	Brain Region (MNI)	Coordinates {mm}			cluster-level		peak-level		
		x	y	z	**k cluster size**	**p-value** (FWE-corr.)	***t***	***Z***	**p-value** (FWE-corr.)
**rCBF Placebo > Psilocybin**									
1	**Postcentral Gyrus L**	-30	-34	56	10579	< 0.000	8.15	6.8	< 0.000
	Inferior Parietal Lobule L	-30	-58	52			7.72	6.53	< 0.000
	Postcentral Gyrus L	-38	-22	50			7.66	6.49	< 0.000
2	**Postcentral Gyrus R**	32	-30	56	2993	< 0.000	6.47	5.7	0.001
	Postcentral Gyrus R	28	-30	64			5.85	5.25	0.005
	Postcentral Gyrus R	12	-38	72			5.58	5.05	0.013
3	**Superior Temporal Gyrus R**	64	-14	8	425	0.006	4.55	4.24	0.303
	Rolandic Operculum R	44	-26	16			4.29	4.02	0.543
	Rolandic Operculum R	36	-22	18			4.17	3.93	0.663

Note: rCBF: Relative Cerebral Blood Flow; L: Left, R: Right

Significance threshold set at p<0.05 cluster-level family-wise-error (FWE) for multiple corrections at an initial voxel-level threshold of p (uncorrected)<0.001, and an extent threshold of k=250 voxels.

Table 3: Brain region with decreased absolute cerebral blood flow

Cluster #	Brain Region (MNI)	Coordinates {mm}				peak-level		
		x	y	z	k cluster size	*t*	*Z*	p-value (FWE-corr.)
**aCBF Placebo > Psilocybin**								
1	**Postcentral Gyrus L**	-34	-30	50	24176	8.8	7.18	< 0.000
	Rolandic Operculum L	-38	-34	20		8.62	7.08	< 0.000
	Inferior Parietal Lobule L	-24	-68	44		8.36	6.92	< 0.000
2	**Rolandic Operculum R**	38	-22	20	676	6.58	5.78	0.001
	Rolandic Operculum R	48	-22	16		6.16	5.48	0.003
	Superior Temporal Gyrus R	64	-16	10		5.79	5.21	0.01
3	**Putamen R**	28	-4	14	224	6.39	5.64	0.001
	Putamen R	30	-18	8		5.88	5.28	0.007
	Pallidum R	20	-6	-4		5.67	5.12	0.015
4	**Middle Frontal Gyrus R**	28	16	56	351	6.28	5.56	0.002
	Superior Frontal Gyrus R	20	8	66		6.21	5.52	0.002
	Superior Frontal Gyrus R	22	8	58		5.71	5.15	0.013
5	**Precuneus L**	-14	-48	8	127	6.03	5.39	0.004
	Precuneus L	-24	-52	2		5.55	5.03	0.023

Note: aCBF: Absolute Cerebral Blood Flow; L: Left, R: Right

Significance threshold set at p<0.05, family-wise error-corrected (FWE-corrected) for multiple comparisons at an initial voxel-level threshold of p (FWE-corrected)<0.05 and an extent threshold of k=100 voxels.
